# Validation in the Greek Language of the Patients’ Perception of the Surgical Safety Questionnaire

**DOI:** 10.7759/cureus.69345

**Published:** 2024-09-13

**Authors:** Aikaterini Toska, Athina Lamprou, Maria Saridi, Kyriakos Souliotis, Stella Zetta, Evangelos C Fradelos

**Affiliations:** 1 Laboratory of Clinical Nursing, Department of Nursing, University of Thessaly, Larissa, GRC; 2 Department of Surgery, General Hospital of Corinth, Corinth, GRC; 3 Department of Nursing, University of Thessaly, Assistant Professor, Larisa, GRC; 4 Department of Research, Health Policy Institute, Athens, GRC; 5 Department of Social and Education Policy, University of Peloponnese, Corinth, GRC; 6 Department of Nursing, School of Social Sciences, Hellenic Open University, University of Thessaly, Larissa, GRC

**Keywords:** patients safety, safety questionnaire, safety study, surgical safety, validation study

## Abstract

Introduction

Adverse events in the operating room are considered unintended injuries or harm caused by healthcare rather than the patient's disease, leading to death, disability, or prolonged hospital stays.

Methods

A cross-sectional design was conducted following the random sampling method, on 200 surgical patients. A self-completed questionnaire was used, translated, and adapted into the Greek language.

Results

Most of the sample (57%) was male. The mean age was 67 years, and 52% were elementary school graduates. Upon analysis, significant correlations were observed between the two-administration (p<0.001) facts (intraclass correlation coefficient = 0.567), which reveal that the scale is stable through time. In addition, Cronbach’s alpha had a value of 0.824 suggesting good internal consistency of the scale. Regarding the subscale, the first factor “safety regarding surgical procedure” was found 0.847, the second factor “effective communication and understanding” was 0.792, and finally the third factor “emotional security” was 0.506. A statistically significant difference found between the dimensions of safety in the operating room and the age of the patients was in the factor of emotional security, revealing that patients aged more than 70 were feeling more secure compared to younger patients (198) = 2.374, p=0.019.

Conclusions

The internal consistency of the Patients' Perspectives of Surgical Safety Scale (PPSS), weighted in a sample of the Greek population, is deemed satisfactory. The safety perceptions of surgical patients are at high levels.

## Introduction

The significance of patient safety, an essential component of high-quality care and an independent discipline, was reaffirmed at the Third Global Ministerial Summit on Patient Safety held in Tokyo in April 2018. Patient safety and responsiveness appear to have been universally accepted as key quality dimensions in healthcare [1]. The World Health Organization estimates that "patient harm" is the fourteenth leading cause of the global disease burden [1,2]. In some European countries, the burden of patient harm is comparable to that of chronic diseases (e.g., multiple sclerosis and certain types of cancer). The Organization for Economic Co-operation and Development (OECD) estimates that approximately 15% of hospital expenditure in OECD countries can be attributed to addressing safety issues, as unsafe care not only has a dramatic impact on patients' lives but also imposes a high economic cost on society as a whole [3].

In recent years, healthcare professionals have focused on patient safety issues to prevent adverse events. Adverse events in the operating room are considered unintended injuries or harm caused by healthcare rather than the patient's disease, leading to death, disability, or prolonged hospital stays. Three percent to 17% of patients experience such events during their hospital stay [4-6]. In some studies, the extended hospital stays lasted nine days [7]; in others, it lasted seven months [8]. A significant number of patient injuries result from improper medical management and the provision of substandard care. Patient injuries during hospitalization due to poor medical management consume a significant portion of available hospital resources [9]. Patients’ perspective regarding perioperative safety is crucial and seems to influence the overall experience of hospitalization and their trust in healthcare services [10].

More specifically regarding patients’ perception of safety, results showed that meanings of safety included not only shared trust in a bureaucratic framework of accreditation, accountability, procedural rules, and regulations but were also individual and context-dependent. For patients, safety has been given interpretations such as "ownership of systems and personal interaction between doctor and patient" [11]. In the study by Dixon et al. (2015), patients felt safe, as demonstrated by the score on the questions "I felt safe on the day of surgery" and "Mistakes rarely happen during surgery." In this study, the questionnaire was distributed to 345 patients, with 102 of them (29.5%) responding. Participant demographics, like race, income, and education level, were similar to the overall patient population. Most respondents were female (62.7%), white (77.5%), and had an annual income of <$75,000 (72.6%). Sixty-six percent (66%) of patients showed laterality in their surgical procedure, and only 11.8% of the present procedure was the first surgical procedure [12].

Although patient safety has been studied in Greece, this issue regarding surgical patients is limited. Greek health professionals know the importance of counting surgical instruments and documentation in the gynecological operating room. Despite the recognition of the importance of security measures, there is a critical knowledge gap regarding responsibilities and standardized procedures during implementation. More effective implementation and increased staff awareness of surgical safety protocols and international guidelines are necessary to improve the quality of surgical safety in Greece [13]. However, only specific aspects have been studied, such as satisfaction with anesthesia, with surgical patients in Greece reporting high satisfaction with anesthesia care during the perioperative period [14]. Thus, this study aims to validate the surgical patients’ safety scale in the Greek language and to assess related factors associated with patients’ perception of safety.

## Materials and methods

A cross-sectional design was employed in this study. The sample consisted of 200 general surgery, orthopedic, and urology patients undergoing surgery in a general regional hospital in Greece. Sample selection was done by random sampling while the questionnaires were collected by the self-completion method, and where this was not possible, the completion of the questionnaires was done in the form of an interview by the researcher.

Data were collected from a self-reported questionnaire consisting of two parts. The first part contained demographic and clinical information such as gender, age, marital status, and level of education. The second part consisted of the questionnaire used in the research of Dixon et al. (2015) titled “Patients’ Perspectives of Surgical Safety: Do They Feel Safe? Permission has already been requested and granted by the creators for the use of the questionnaire. The questionnaire has been translated into Greek using the World Health Organization guidelines for translation and validation instruments and adapted according to the current conditions of Greek hospitals.

Statistical analysis

The SPSS statistical program (version 25, IBM Corp., Armonk, NY, US) was used to analyze the data. In addition, JASP 0.15.0.0 (https://jasp-stats.org/previous-versions/) was used for confirmatory factor analysis (CFA). Processing was based on descriptive statistics in percentage distributions, mean values, and standard deviations, as well as inductive statistics on students' t-tests and analysis of variances, depending on the data. In addition, exploratory and confirmatory factor analysis was performed to assess the construct validity of the scale. The level of statistical significance will be set at 0.05.

Ethical issues

Permission was obtained before the study from the scientific committee of the General Hospital of Corinth (approval number: 24351/1-10-2019). Completing the questionnaire was voluntary. The anonymity of the study participants was maintained, and they had the option to withdraw at any stage of the research. There was no financial burden on the hospital, and the results were used exclusively for the study.

## Results

The majority of the sample (57%) was male. The mean age of the sample was 67 years. Fifty-two percent (52%) of the patients were elementary school graduates and 20.5% had completed high school. Sixty-seven point five percent (67.5%) were married. Thirty-nine point five percent (39.5%) of the sample stated that they performed surgery, 36.5% orthopedic surgery, and 24% urological surgery. Detailed information regarding the characteristics of the sample is presented in Table 1.

**Table 1:** Sociodemographic and clinical characteristics of the sample

	Number	Rate
Gender		
Male	114	57,0
Female	86	43,0
Age group		
<70 years old	97	48,5
>71years old	103	51,5
Age: mean: 67 (±18,6)		
Educational level		
Elementary school	104	52,0
Gymnasium	25	12,5
High school	41	20,5
Vocational Training Institute (ΙΕΚ )	8	4,0
University	20	10,0
Postgraduate title	2	1,0
Family status		
Married	135	67,5
Unmarried	24	12,0
Divorced	12	6,0
Widowed	29	14,5
Type of treatment		
Orthopedic	73	36,5
Surgical	79	39,5
Urological	48	24,0

Table 2 shows the descriptive analysis of questions about operating room safety. The higher value was observed in the tenth question “Safety was the most important part of the surgical procedure” while the lowest value was observed in the fifteenth question “I understood the risks associated with surgery”.

**Table 2:** Descriptive analysis of questions related to safety in the operating room

	N	min	max	mean	SD
Item 1	200	1.00	7.00	5.98	1.17
Item 2	200	2.00	7.00	4.86	1.09
Item 3	200	3.00	7.00	5.80	0.75
Item 4	200	2.00	7.00	5.76	0.75
Item 5	200	3.00	7.00	5.66	0.78
Item 6	200	3.00	7.00	5.64	0.69
Item 7	200	1.00	7.00	4.76	1.11
Item 8	200	2.00	7.00	4.99	0.92
Item 9	200	2.00	7.00	4.74	1.05
Item 10	200	4.00	7.00	6.03	0.58
Item 11	200	3.00	7.00	5.07	1.03
Item 12	200	2.00	7.00	5.38	0.86
Item 13	200	2.00	7.00	5.43	0.89
Item 14	200	2.00	7.00	5.26	1.40
Item 15	200	1.00	7.00	3.62	1.73
Item 16	200	1.00	7.00	4.95	1.57
Item 17	200	1.00	7.00	4.83	1.59
Item 18	200	2.00	7.00	5.26	1.00
Item 19	200	2.00	7.00	5.67	1.14
Item 20	200	1.00	7.00	4.49	1.41
Total Score	200	2.71	6.51	5.18	0.67

Construct validity of patients’ perspectives of surgical safety scale

Exploratory and confirmatory factor analyses were applied to examine the factorial structure of the patients' perspectives of surgical safety scale questionnaire (PPSSQ). In the first step, the Kaiser-Meyer-Olkin (KMO) statistic and Bartlett’s sphericity test were performed to examine the sample’s adequacy for factor analysis. The value of KMO 0.884, x2 = 1610.257, and the significance of the Bartlett test (p<0.001) indicated that data were adequate for factor analysis. Exploratory factor analysis was performed with principal component analysis to identify the factors of the scale. The appropriate number of factors was determined by eigenvalues greater than 1. The EFA resulted in three three-factor solutions for the scale interpreting 49.3% of the variance, which differs from the original version. Finally, we performed a CFA to test the three-factor structure of the scale. Regarding CFA, the model tested was equivalent to other proposed structures of the Patients’ Perspectives of Surgical Safety Scale. The model presented a reasonably good fit to the data. The chi-square/df ratio was 1.9, less than 3.00 indicating an acceptable fit, Tucker-Lewis index (TLI) was 0.88, the comparative fit index (CFI) was close to 0.89 and the root mean square error of approximation (RMSEA) was 0.074 and lower than 0.10. Overall, our CFA confirmed the three-factor structure of the scale (Table 3).

**Table 3:** Exploratory and confirmatory factor analysis results Note: The applied rotation method was Promax.

	Factor 1	Factor 2	Factor 3	Item to Total Correlation	Kaiser-Meyer-Olkin test
	Safety regarding surgical procedure	Effective communication and understanding	Emotional security	Pearson r coefficient	Overall MSA= 0.884
Q1	0.447	0.369		0.354	0.962
Q2		0.629		0.333	0.908
Q3	0.784			0.271	0.926
Q4	0.790			0.431	0.921
Q5	0.840			0.359	0.875
Q6	0.884			0.328	0.872
Q7		0.833		0.516	0.875
Q8		0.742		0.435	0.892
Q9		0.780		0.405	0.900
Q10	0.665			0.257	0.909
Q11		0.464		0.316	0.952
Q12		0.536		0.652	0.874
Q13		0.528		0.667	0.872
Q14		0.483		0.286	0.853
Q15		0.507		0.234	0.762
Q16			0.610	0.565	0.566
Q17			0.769	0.569	0.504
Q18			0.322	0.568	0.892
Q19			0.599	0.559	0.817
Q20			0.449	0.210	0.753
Mean (SD)	34.8(3.6)	44.0(6.3)	25.1(3.9)	5.18(0.76)	
Cronb.a	0.847	0.792	0.506	0.824	

Reliability analysis of the patients’ perspectives of surgical safety scale

The test-retest method was applied to explore the test-retest reliability of the patients’ perspectives of surgical safety scale. A purposeful sample of 30 patients completed the questionnaire at baseline and two weeks later. This is estimated as adequate time so that the individuals do not recall their answers [15]. Upon the analysis, significant correlations were observed between the two-administration (p<0.001) facts (Intraclass Correlation Coefficients = 0.567) that reveal that the scale is stable through time. In addition, Cronbach’s alpha had a value of 0.824 suggesting good internal consistency of the scale. Regarding the subscale, the first factor “Safety regarding surgical procedure” was found 0.847, the second factor “Effective communication and understanding” was 0.792, and finally the third factor “Emotional security” was 0.506 (Table 3). Moreover, the value of Cronbach’s Alpha didn't increase substantially if any item was deleted from the scale. All items exhibited strong correlations to the total score. This fact adds to the excellent internal consistency of the scale.

The only statistically significant difference found between the dimensions of safety in the operating room and the age of the patients was in the factor of emotional security, revealing that patients aged more than 70 were feeling more secure compared to younger patients (198) = 2.374, p=0.019. Thus, we can say that people <70 years old and people >70 years old felt the same safety in the operating room. Detailed information is presented in Table 4.

**Table 4:** Differences in scale score regarding age

	Group	Mean	Standard Deviation	t-test	Degrees of Freedom	p-value
Safety regarding surgical procedure	<70 Years	35.20	3.4	1.329	198	0.186
	>70 Years	34.52	3.7			
Effective communication and understanding	<70 Years	44.86	6.4	1.675	198	0.095
	>70 Years	43.36	6.2			
Emotional security	<70 Years	25.86	3.7	2.374	198	0.019
	>70 Years	24.55	4.0			

## Discussion

This research aims to evaluate the Greek version of the PPSSQ and to investigate the patients’ perceptions regarding safety in the operating room. The PPSSQ was weighted in a sample of the Greek population in terms of age and educational level, which consisted of patients who had undergone surgical procedures. The internal consistency of the PPSS is considered satisfactory (Cronbach's alpha 0.824). This finding is consistent with previous studies in which internal reliability was satisfactory in a representative sample of American [12] and Norwegian surgical patients [16]. The PPSS was initially proposed as a questionnaire with four dimensions [12]; however, later studies suggested another structure for the questionnaire [16]. In the present study, the EFA highlighted the choice of interpreting the results based on three dimensions: safety regarding surgical procedure, effective communication and understanding, and emotional security. Such differences in factorial structures are often observed in validation and cultural adaptation studies highlighting different perceptions through cultures.

In addition, surgical patients overwhelmingly stated feeling safe during the pre-operative, recovery period, and human interactions. The results showed that the perceived good care causes feelings of security and high levels of satisfaction in patients. A significant finding was patients’ perception that surgical safety was a priority of the surgical team members. However, patients were not aware of the measures and procedures implemented to improve surgical safety, such as surgical checklists.

The results of the present research regarding patient safety conditions agree with the international literature and specific research by Moser et al. (2007) [17] and Lantz et al. (2005) [18] and state that the choice of safety conditions is related to the basic principles of quality care, including a good patient-medical staff relationship. Also, safety in the operating room as a predictor of good care is consistent with a study by Joffe et al. (2003) where patients' experiences were associated with their willingness to recommend the hospital [19]. The survey showed that a significant percentage of patients agreed that the surgical team's main priority was their safety. In particular, the patients feel safe when, on the day of surgery, all the surgical team members are introduced to them and informed about their role in the surgical procedure. Similar research by Aouicha et al [20]. (2022) and Wami et al [21]. (2016) report that patient perceptions about safety are influenced by information sharing among the surgical team, and the information to patients about safety practices could bridge the gap between patient perceptions of safety and reality. Thus, the level of communication in all phases of care could have a significant impact on hospital quality metrics.

Our study showed that patients' perceptions of safety are not influenced by sociodemographic characteristics, such as gender or marital status, a finding that agrees with those of Dixon et al. (2015). Furthermore, we did not find a statistically significant difference between safety dimensions in the operating room and the educational level of the patients [12], given that despite a large percentage of patients being primary school graduates, they felt as safe as patients with a higher educational level. Conversely, similar studies abroad report that patients with a lower level of education were more likely to feel unsafe in the hospital environment and be less satisfied with the care provided [22,23].

The innovation of this study is manifested in some distinct aspects. It is the first study in Greece that utilizes the PPSSQ to assess patient’s perception of safety. In addition, this research systematically explores the social and demographic factors that are influencing patient’s perception of safety. Our study has some limitations that do not allow the generalization of the results. First, the representativeness of the sample is limited and only from one hospital. In addition, the cross-sectional study design allows the examination of changes in the perception of safety during hospitalization.

## Conclusions

The internal consistency of the PPSS, weighted in a sample of the Greek population that had undergone surgical procedures, is deemed satisfactory. Safety perceptions of surgical patients are at high levels without being influenced by sociodemographic characteristics such as gender, age, family status, or educational level. Good care and communication, in all stages of surgical care, cause feelings of trust, security, and high levels of satisfaction in patients, as well as the sense that their safety is the main priority of the surgical team, despite the fact they are not informed about the measures taken and the procedures implemented to improve their safety such as surgical checklists. This shows a gap in the information procedure, which leads to the need to provide specific information to patients about the safety measures undertaken to optimize comfort and the sense of safety in the operating theater.

## References

[1] Busse R, Klazinga N, Panteli D, Quentin W (2019). Improving healthcare quality in Europe: characteristics. effectiveness and implementation of different strategies. https://iris.who.int/bitstream/handle/10665/327356/9789289051750-eng.pdf?sequence=1&isAllowed=y.

[2] Dhingra-Kumar N. Brusaferro S. Arnoldo L Patient safety in the world. Textbook of Patient Safety and Clinical Risk Management.

[3] Slawomirski L. Auraaen A. Klazinga N (2017). The economics of patient safety. OECD Health Working Papers.

[4] Baker GR, Norton PG, Flintoft V (2004). The Canadian Adverse Events Study: the incidence of adverse events among hospital patients in Canada. CMAJ.

[5] San Jose-Saras D, Valencia-Martín JL, Vicente-Guijarro J, Moreno-Nunez P, Pardo-Hernández A, Aranaz-Andres JM (2022). Adverse events: an expensive and avoidable hospital problem. Ann Med.

[6] Zanetti AC, Dias BM, Bernardes A (2021). Incidence and preventability of adverse events in adult patients admitted to a Brazilian teaching hospital. PLoS One.

[7] Davis P, Lay-Yee R, Briant R, Ali W, Scott A, Schug S (2002). Adverse events in New Zealand public hospitals I: occurrence and impact. N Z Med J.

[8] Schiøler T. Lipczak H. Pedersen BL. Mogensen TS. Bech KB. Stockmarr A. Svenning AR. Frølich A; Danish Adverse Event Study (2001). Incidence of adverse events in hospitals. A retrospective study of medical records [Article in Danish]. Ugeskr Laeger.

[9] Soop M, Fryksmark U, Köster M, Haglund B (2009). The incidence of adverse events in Swedish hospitals: a retrospective medical record review study. Int J Qual Health Care.

[10] Larsson F, Strömbäck U, Rysst Gustafsson S, Engström Å (2023). Perception of feeling safe perioperatively: a concept analysis. Int J Qual Stud Health Well-being.

[11] Rhodes P, Campbell S, Sanders C (2016). Trust, temporality and systems: how do patients understand patient safety in primary care? A qualitative study. Health Expect.

[12] Dixon JL. Tillman MM. Wehbe-Janek H. Song J. Papaconstantinou HT (2015). Patients’ perspectives of surgical safety: do they feel safe?. Ochsner J.

[13] Dinas K, Vavoulidis E, Pratilas GC (2019). Gynecology healthcare professionals towards safety procedures in operation rooms aiming to enhanced quality of medical services in Greece. Int J Health Care Qual Assur.

[14] Kouki P, Matsota P, Christodoulaki K (2012). Greek surgical patients' satisfaction related to perioperative anesthetic services in an academic institute. Patient Prefer Adherence.

[15] WHOQOL translation methodology. http://www.who.int/docs/default-source/publishing-policies/whoqol-100-guidelines/translation-methodology.pdf.

[16] Husebø SE, Olden M, Pedersen M, Porthun J, Balllangrud R (2023). Translation and psychometric testing of the Norwegian version of the “patients’ perspectives of surgical safety questionnaire”. J Perianesth Nurs.

[17] Moser A, Houtepen R, Widdershoven G (2007). Patient autonomy in nurse-led shared care: a review of theoretical and empirical literature. J Adv Nurs.

[18] Lantz PM, Janz NK, Fagerlin A (2005). Satisfaction with surgery outcomes and the decision process in a population-based sample of women with breast cancer. Health Serv Res.

[19] Joffe S, Manocchia M, Weeks JC, Cleary PD (2003). What do patients value in their hospital care? An empirical perspective on autonomy centred bioethics. J Med Ethics.

[20] Aouicha W, Tlili MA, Sahli J (2022). Patient safety culture as perceived by operating room professionals: a mixed-methods study. BMC Health Serv Res.

[21] Wami SD, Demssie AF, Wassie MM, Ahmed AN (2016). Patient safety culture and associated factors: a quantitative and qualitative study of healthcare workers' view in Jimma Zone Hospitals, Southwest Ethiopia. BMC Health Serv Res.

[22] Hamilton T, Macki M, Oh SY (2022). The association of patient education level with outcomes after elective lumbar surgery: a Michigan Spine Surgery Improvement Collaborative study. J Neurosurg Spine.

[23] De Oliveira GS Jr, McCarthy RJ, Wolf MS, Holl J (2015). The impact of health literacy in the care of surgical patients: a qualitative systematic review. BMC Surg.

